# Complete manuscript Title: Changes in RANKL during the first two years after cART initiation in HIV-infected cART naïve adults

**DOI:** 10.1186/s12879-017-2368-y

**Published:** 2017-04-11

**Authors:** Inger Hee Mathiesen, Mohammad Salem, Jan Gerstoft, Julie Christine Gaardbo, Niels Obel, Court Pedersen, Henrik Ullum, Susanne Dam Nielsen, Ann-Brit Eg Hansen

**Affiliations:** 1 Department of Infectious Diseases, Copenhagen University Hospital Rigshospitalet, Copenhagen, Denmark; 2 Department of Microbiology & Immunology, Hollings Cancer Center, Medical University of South Carolina, Charleston, SC USA; 3 Department of Infectious Diseases, Odense University Hospital, Odense, Denmark; 4 Department of Clinical Immunology, Copenhagen University Hospital Rigshospitalet, Copenhagen, Denmark; 5 Department of Infectious Diseases, Copenhagen University Hospital Hvidovre Hospital, Kettegaard Allé 30, -2650 Copenhagen, DK Denmark

**Keywords:** RANKL, OPG, cART, BMD, HIV infection

## Abstract

**Background:**

By assessing the changes in concentration of soluble receptor activator of nuclear factor κ B ligand (RANKL) and osteoprotegrin (OPG) after initiation of combination antiretroviral therapy (cART) in treatment-naïve HIV-infected patients we aimed to evaluate whether the initial accelerated bone loss could be mediated by increased soluble RANKL (sRANKL) levels associated with CD4+ T cell recovery.

**Methods:**

We used multiplex immunoassays to determine sRANKL and OPG concentrations in plasma from 48 HIV patients at baseline and 12, 24, 48 and 96 weeks after cART initiation.

**Results:**

Soluble RANKL changed significantly over time (overall *p* = 0.02) with 25% decrease (95% CI: -42 to −5) at week 24 compared to baseline and stabilized at a lower level thereafter. We found no correlation between CD4+ T cell count increment and changes in sRANKL or between percentage change in BMD and changes in sRANKL.

**Conclusion:**

In this study there was no indication that the accelerated bone loss after cART initiation was mediated by early changes in sRANKL due to CD4+ T cell recovery. Future studies should focus on the initial weeks after initiation of cART.

**Trial registration:**

Clinical-Trial.gov. id NCT00135460, August 25, 2005. The study was approved by the Danish Data Protection Agency, Danish Medicines Agency and Regional Ethics Committee.

## Background

HIV-infected patients experience a loss in bone mineral density (BMD) during the first 6–12 months after initiation of combination antiretroviral therapy (cART) [1–5]. The bone loss associated with cART initiation has been observed for all combinations of drug classes [6] although Tenofovir disoproxil fumarate (TDF) containing regimens are associated with more pronounced decreases in BMD of an additional 1.4%–2.0% [3, 7]. The molecular mechanisms behind the accelerated bone loss are not fully understood.

A key system in bone turnover is the RANK/RANKL/OPG system [8]. Receptor activator of Nuclear Factor κ B(RANK) expressed on osteoclast surfaces binds receptor activator of nuclear factor κ B ligand(RANKL), which is expressed on osteoblast surfaces but also secreted by endothelial cells, B lymphocytes and T lymphocytes [9]. Binding of RANKL to RANK initiates a cascade of intracellular pathways which activates gene transcription necessary for osteoclast maturation and activity [9]. Osteoprotegrin (OPG) is produced by osteoblasts and works as a decoy receptor of RANKL thus inhibiting osteoclast activity [9].

A study of HIV-1 transgenic rats reported increased bone degradation and a shift in the ratio of RANKL to OPG favouring osteoclastogenesis and hence bone loss [10], findings which were later validated in HIV positive subjects [11]. A further study in T-cell depleted knockout mice demonstrated that transfusion of T-cells corresponded to increased RANKL concentrations and bone loss (BMD evaluated by DXA scan) within the repleted mice [12]. In other inflammatory conditions such as chronic colitis, activated CD4 + T cells have been shown to produce RANKL [13]. These data suggest that the immune reconstitution and/or CD4 + T cell recovery after cART initiation could be a contributing factor to bone loss. In a previous study [1], we have shown a significant bone loss after cART initiation at the spine and femoral neck after 24 and 48 weeks, respectively. Furthermore, more pronounced bone loss was found in individuals with very low CD4 + T cell count at time of cART initiation. Other prospective studies have also found that low baseline CD4 + T cell count [3], especially CD4 + T cell count below 50 cells/mm^3^ [14, 15], was a predictor of more pronounced bone loss after cART initiation.

In the present study, we aimed to assess the changes in soluble RANKL (sRANKL) and OPG repeatedly over 96 weeks in treatment-naïve HIV-infected patients initiating cART. We hypothesized that treatment with cART would result in increased sRANKL concentration and altered RANKL/OPG ratio during the first months after cART initiation due to CD4 + T cell recovery. We also hypothesized that the early increase in sRANKL concentrations would be negatively correlated with baseline CD4 + T cell count. Finally, we hypothesized that sRANKL and RANKL/OPG ratio would correlate negatively with BMD.

## Methods

### Study population

The SPAR BMD study has been described previously [1]. In this study patients from three Danish centers specialized in HIV treatment were randomized to a protease inhibitor (PI) sparing regimen (zidovudine/lamivudine/efavirenz) or a nucleoside reverse transcriptase inhibitor (NRTI) sparing regimen (lopinavir/ritonavir/efavirenz). Site specific Dual X Ray Absorptiometry (DXA) scans were performed to evaluate spine and femoral neck BMD at baseline and week 24, 48, 96 and 144 after initiation of cART. In the present study, we used stored plasma samples from patients from the two largest centers to measure sRANKL and OPG at baseline and week 12, 24, 48 and 96. Three patients received oral corticosteroids during the study period; two patients within the first study year, and one patient within the second study year. No patients received bisphosphonates or other anti-osteoporotic medication during the study period.

### Measurement of soluble RANKL and OPG

At baseline, week 12, 24, 48 and 96 a 10 ml EDTA blood sample (non-fasting) was drawn. Samples were immediately centrifuged for 10 min at 3000 g at 0°-4° Celsius, then plasma was transferred to Nunc™ tubes and frozen immediately at −80° Celsius until analysis.

Concentration of sRANKL and OPG were determined by multiplex analysis (Biomedica, Vienna, Austria) according to the manufacturer’s instructions. For RANKL, 100 μl of pre-prepared standards/controls and samples were added to the reaction wells of the microarray, and 100 μl biotinylated anti soluble RANKL antibody was then added to each well. The plate was incubated over night at room temperature with continuous shaking. The following day the antibody-coupled microspheres were washed 5 times in pre-prepared wash buffer and incubated with 200 μl streptavidin-alkaline phosphate for 1 h at room temperature. Then the plate was washed as above and 100 μl of each amplifier A (inorganic salts and buffered enzyme solution with tetrazolium violet) and amplifier B (stabilized NADPH solution) per well were added and again incubated at room temperature for 45 min. Finally, 50 μl stop solution was added to each well and the absorbance was measured immediately at 490 nm with reference 630 nm.

For OPG, 100 μl assay buffer, 50 μl standards/controls/samples, and 50 μl biotinylated anti OPG antibody was mixed, added to plate, and incubated over night at room temperature with continuous shaking. After incubation, antibody-coupled microspheres were washed 5 times in wash buffer and incubated with 200 μl assay substrate for 20 min at room temperature. The reaction was then stopped as before and the absorbance was measured immediately at 450 nm with reference 630 nm. According to the manufacturer the inter-assay CV was <15% and intra-assay CV was <10% for both RANKL and OPG. All reads were performed using a Luminex 100 System (Luminex 100™ platform (Luminex Corp., Austin, TX, USA) and samples from the same participant were run on the same plate.

### Statistical analyses

Baseline characteristics and measurements of sRANKL, OPG concentrations, CD4 + T cells and BMD and T scores were expressed as medians and interquartile range (IQR) or mean and standard deviation (SD) as appropriate. Differences between groups were compared using Mann Whitney U test and χ ^2^ test as appropriate. OPG, sRANKL and OPG/RANKL ratio were log2 transformed, and CD4 + T cell count was square rooted to obtain normal distribution. A linear mixed model (LMM) was applied to analyze mean changes in sRANKL and OPG concentrations over time and to analyze whether treatment group was associated with changes in sRANKL or OPG over time. LMM data was backtransformed and calculated as percent change from baseline. We used Student’s T test to compare changes in sRANKL from baseline (ΔRANKL) at week 12 and 24 in patients with baseline CD4 + T cell count <200 cells/μl versus patients with baseline CD4 + T cell count ≥200 cells/μl. Pearson’s correlation was used to evaluate correlations between CD4 + T cell increment (ΔCD4) and ΔRANKL difference from baseline to week 12. Associations between baseline RANKL/OPG ratio and BMD and percentage change in BMD and ΔRANKL at week 12 were also analyzed. Multivariate linear regression was used to adjust for age, sex and BMI at baseline. Statistical analysis was performed with IBM SPSS Statistics 22. A *p* value ≤0.05 was considered significant.

## Results

A total of 48 patients with baseline BMD and RANKL/OPG measurements and at least one follow-up BMD and RANKL/OPG measurement participated in the BMD RANKL sub study (23 in the NRTI sparing and 25 in the PI sparing group). There were no differences in baseline characteristics (Table 1) and therefore groups were pooled in the following analyses.

**Table 1:** Baseline characteristics

Entry	Overall	NRTI sparing	PI sparing	*P* level
No	48	23	25	-
Male, (%)	44 (92)	21 (91)	24 (96)	0.90
Age (years)	41.9 (38.12–53.72)	41.30 (38.2–50.9)	41.90 (38.1–54.6)	0.67
Weight (kg)	70.95 (65.17–80.86)	74 (66–88.5)	69.75 (64.4–73.5)	0.06
Height (cm)	178 (173.5–184.75)	178 (172.5–182)	178 (174–186)	0.71
BMI (kg/cm^2^)	22.03 (20.48–24.94)	23.92 (21.3–25.76)	21.65 (20.46–22.84)	0.06
CD4 + T cell count baseline (cells/μl)	200 (86–280)	240 (110–315)	170 (70–280)	0.20
CD4 + T cell count nadir (cells/μl)	160 (86–250)	200 (110–250)	140 (68–240)	0.21
Spine BMD (g/cm^2^)	1.09 (0.926–1.20)	1.11 (0.96–1.22)	1.04 (0.96–1.18)	0.38
Spine T-score	−0.34 (−1.70–0.71)	−0.15 (−1.36–0.83)	−0.71 (−1.78–0.40)	0.35
Spine Z-score	−0.01 (−1.34–0.85)	0.00 (−0.64–1.2)	−0.12 (−1.3–0.74)	0.41
Hip BMD (g/cm^2^)	0.89 (0.78–0.96)	0.90 (0.81–0.95)	0.86 (0.78–0.96)	0.70
Hip T-score	−0.89 (−1.67 - -0.34)	−0.86 (−1.5 - -0.37)	−0.96 (−1.63 - -0.44)	0.78
Hip Z-score	−0.34 (−0.86–0.42)	−0.31 (−0.62–0.32)	−0.37 (−0.97–0.31)	0.63
HIV RNA (log10 copies/ml)	5.24 (4.69–5.47)	5.01 (4.5–5.7)	5.30 (5.1–5.5)	0.37
RANKL (pg/ml)	7.79 (2.83–12.01)	8.72 (2.79–12.93)	7.38 (2.8–11-93)	0.83
OPG (pg/ml)	34.19 (26.53–45.75)	37.73 (27.95–45.92)	32.08 (24.19–51.72)	0.93
RANKL/OPG ratio	0.21 (0.08–0.36)	0.18 (0.1–0.38)	0.25 (0.07–0.36)	0.82

Continuous data expressed as median and interquartile range (IQR), nominal data expressed as frequency (%)

*μl* microliter, *kg* kilograms, *cm* centimeters, *g* grams, *pg* picograms

Evolution of BMD over 144 weeks in the randomized trial has been described in details previously [1]. Major findings were a marked decrease in BMD at the spine and femoral neck at week 24 and 48. In the present substudy, the percentage change from baseline at the lumbar spine was −2.86 (95% CI; −3.73 to −1.99) at week 24, and −2.26 (95% CI; −3.39 to −1.14) at week 48. The percentage change from baseline at the femoral neck was −2.59 (95% CI; −4.61 to −1.57) at week 24 and −5.01 (95% CI; −6.42 to −3.58) at week 48. All changes were statistically significant (*p* < 0.001). There were no significant differences between the PI-sparing arm and the NRTI-sparing arm.

### Evolution of RANKL and OPG during cART treatment

Over time, sRANKL changed significantly (overall *p* = 0.02) with a decline and stabilization from week 24 and onwards.

The mean estimated percent change in sRANKL from baseline was 2% at week 12 (95% CI: -20 to 31), −25% (95% CI: -42 to −5) at week 24, −20% (95% CI: -37 to 4) at week 48 and −27% (95% CI: -45 to −5) at week 96.

OPG levels did not change significantly over time (overall *p* = 0.32) although there was a trend towards an increase from baseline to week 12. The mean estimated change from baseline was 12% (95% CI: 1–24) at week 12, 5% (95% CI: -6 to 16) at week 24, 8% (95% CI: -3 to 21) at week 48, and 8% (95% CI: -3 to 22) at week 96. Moreover, RANKL/OPG ratio decreased over time, but the changes were not statistically significant. There was no association between treatment group and changes in sRANKL over time (data not shown).

**Fig. 1:**
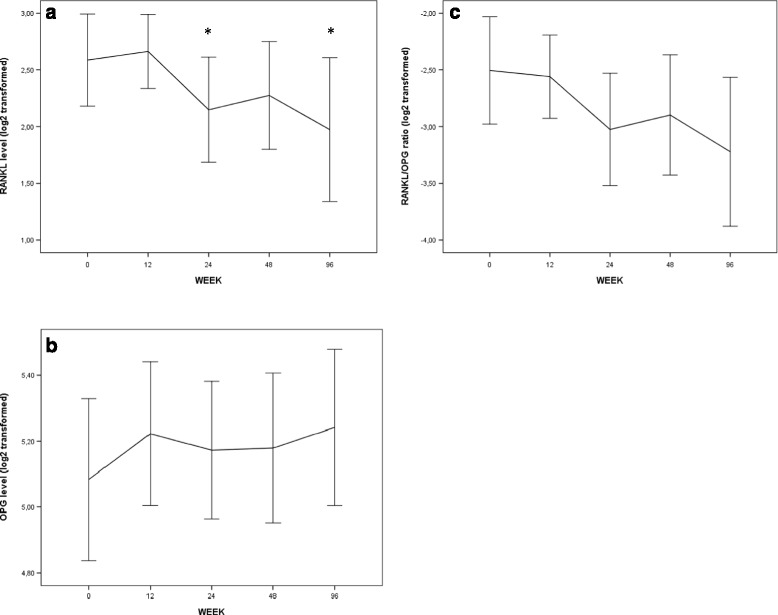
Overall mean RANKL, OPG and RANKL/OPG ratio. Mean levels (95% CI) of (1**a**) RANKL, (1**b**) OPG and (1**c**) RANKL/OPG ratio. Data are logarithmically transformed. 1 unit change in log2 unit corresponds to a doubling in original unit. *p < 0.05 compared to baseline value

The mean levels of sRANKL, OPG and RANKL/OPG ratio are depicted in Table 2 and Fig. 1.

**Table 2:** RANKL, OPG and CD4+ T cell count in overall cohort

WEEK	sRANKL	sOPG	RANKL/OPG ratio	CD4 + T cells
week 0	7.79 (2.83–12.01) (*n* = 48)	34.19 (26.53–45.75) (*n* = 48)	0.21 (0.08–0.36)	200 (86–280) (*n* = 48)
week 12	8.03 (3.72–11.01) (*n* = 45)	40.52 (28.55–53.67) (*n* = 45)	0.18 (0.11–0.30)	300 (202–400) (*n* = 48)
week 24	5.75 (2.88–9.41) (*n* = 45)	35.83 (25.54–50.66) (*n* = 45)	0.17 (0.07–0.29)	340 (240–470) (*n* = 47)
week 48	5.92 (3.07–11.98) (*n* = 43)	36.10 (26.19–55.82) (*n* = 42)	0.16 (0.09–0.35)	360 (250–535) (*n* = 45)
week 96	6.03 (1.02–11.31) (*n* = 34)	38.24 (29.82–50.47) (*n* = 33)	0.15 (0.05–0.30)	450 (280–600) (*n* = 43)

Values are depicted as median and interquartile range (IQR). sRANKL and OPG in picograms/ml (pg/ml) and CD4+ T cells as cells/μl

### CD4+ T cell count and sRANKL concentrations

CD4 + T cell counts are depicted in Table 2. ΔRANKL at week 12 and week 24 did not differ significantly in the group of patients with CD4 + T cells below 200 cells/μl (*n* = 25) versus patients with CD4 + T cells above 200 cells/μl (*n* = 23) at baseline in crude analysis and when adjusted for body mass index(BMI), gender, and age (data not shown). Neither was there any correlation between CD4 + T cell increment and ΔRANKL at week 12(*r* = 0.039, *p* = 0.81).

### RANKL/OPG ratio, sRANKL increment and association with BMD at baseline and week 24

Baseline RANKL/OPG ratio was not significantly correlated with baseline spine or femoral neck BMD, (*r* = 0.04 *p* = 0.78 and *r* = 0.13 *p* = 0.41, respectively). This remained insignificant after adjusting for BMI, gender and age in multivariate analysis (data not shown). There was no correlation between week 12 sRANKL increment and percentage changes in BMD at week 24 at the lumbar spine (*r* = 0.007 *p* = 0.96) or at the femoral neck (*r* = 0.059 *p* = 0.71), also not in analyses adjusted for age, BMI and gender.

## Discussion

In the present study, we measured sRANKL and OPG at multiple time points within the first two years after cART initiation in treatment-naïve HIV-infected patients. sRANKL values had decreased at week 24 and stabilized at a lower level thereafter. The decrease might help to explain the stagnation of cART related bone loss after 6–9 months of therapy, as a decline in RANKL levels leads to decreased osteoclast activation. Similar results with a decline in RANKL levels 6 to 12 months after initiation of cART have been reported in other studies [16, 17].

In contrast to our hypothesis, we did not detect a significant increase in sRANKL at week 12 and our results do not explain the accelerated BMD loss occurring after cART initiation. Possibly, changes in soluble RANKL important for the accelerated bone loss may have taken place already before week 12, since it is well established that bone turnover, measured as increasing levels of C-terminal-telopeptide-cross-links (CTx, a marker of bone degradation and thereby osteoclast activity), and osteocalcin (OC, a protein secreted by mineralizing osteoblasts), is accelerated as early as two weeks after commencing cART [16, 18]. The observed non-significant decrease in sRANKL to OPG ratio could reflect that we captured the resetting of bone metabolism after a catabolic window within the first weeks after cART initiation. However, recently published data [18] did not display any increase in plasma RANKL at week 2, where a marked increase in bone resorption markers had already taken place, indicating that sRANKL may not be the main mediator of bone loss. This is further underscored by the fact that BMD loss at week 24 did not correlate with sRANKL increment at week 12 in our study. Combining these results it seems, that the mechanism of cART induced bone loss was not linked directly to changes in sRANKL.

Indeed, it has been suggested, that the normal regulation of bone resorption and bone formation mediated by RANK/RANKL/OPG, is uncoupled in HIV treatment-naïve patients in favour of increased osteoclast activity [16]. This will, when bone turnover is increased with cART initiation, lead to the observed accelerated bone loss. Another explanation of the lack of association between sRANKL and BMD loss could be, that sRANKL may not reflect what takes place in the bone microenvironment i.e. direct cross talk between membrane bound RANKL in osteoblasts and membrane bound RANK in osteoclasts, which may be of greater importance of osteoclast activation than soluble RANKL [19]. Unfortunately we did not have any bone turnover markers in order to evaluate bone turnover in context of the observed BMD reductions.

Despite animal models having shown a positive correlation between increasing CD4 + T cell count and sRANKL and BMD loss [12], we did not find any association between changes in sRANKL and baseline CD4 + T cell count or magnitude of CD4 + T cell recovery in our present study. In contrast, a recently published study observed significantly increased RANKL levels 12 and 24 weeks after cART initiation coinciding with CD4 + T cell recovery in a group of patients initiating cART (*n* = 19, 18]. These diverting results may be attributable to several differences in study design including study population size (*n* = 48 vs *n* = 19), difference in ethnic origin and a TDF-containing regimen in the study by Ofotokun *et al*. We also had a lower proportion of patients with very low CD4 cell counts. In our study, only five patients (10%) had baseline CD4 + T cell count below 50 cells/μl, which may have hampered the power to find an association between CD4+ T cell reconstitution, changes in sRANKL and changes in BMD.

The major strength of this prospective study is that we repeatedly measured sRANKL within the two first years after cART initiation *including* measurements at week 12 and 24, the period where the accelerated bone loss induced by cART initiation peaks. Only one other group has successfully measured sRANKL levels at multiple time points within 24 weeks of cART initiation [18]. In our study, the patients were randomized to a cART regimen with or without NRTIs. Although the regimens are less modern regimens, the design ensured that we were evaluating the immunological effect of treatment initiation rather than the direct drug effect of NRTIs or TDF.

Our study has several limitations. The study population is relatively small and there is a risk of type II error given the large biological variability of sRANKL [18]. We did not measure any proresorptive cytokines such as Tumor Necrosis Factor-alpha, interleukin-1 or interleukin-6 or any bone turnover markers which would help characterizing the role of sRANKL on bone turnover just after cART initiation. The degree of cross validation between different RANKL immune and ELISA assays are not well established making it difficult to directly extrapolate results between studies [20]. Further, blood sampling in this study was not performed fasting, which may affect within subject variability; and we only measured sRANKL and not RANKL expression by B-cells, other immune cells or osteoblasts. Finally, the majority of participants were males, and findings may not apply to women.

## Conclusion

In summary, we found that sRANKL decreased to levels below baseline values from week 24 and onwards after initiation of cART. In our study, in which median baseline CD4 + T cell count was around 200 cells/μl, there was no indication that the early cART induced accelerated bone loss was mediated through increased levels of sRANKL induced by CD4+ T cell recovery. Further studies focusing on the initial weeks after initiation of cART are warranted.

## Abbreviations

μl: Microliter
ml: Milliliter
kg: Kilograms
g: Grams
pg: Picograms
cm: Centimeters
BMD: Bone mineral density
DXA-scan: Dual X ray absorptiometry scan
sRANKL: Soluble receptor activator of nuclear factor κ B ligand
OPG: Osteoprotegrin
cART: Combination antiretroviral therapy
PI: Protease inhibitor
NRTI: Nucleoside reverse transcriptase inhibitor
TDF: Tenofovir disoproxil fumarate
CTx: C-terminal telopeptide cross links.
OC: Osteocalcin
